# Three Criteria for Ecological Fallacy

**DOI:** 10.1289/ehp.1103768

**Published:** 2011-08-01

**Authors:** Alvaro J. Idrovo

**Affiliations:** Center for Health Systems Research, National Institute of Public Health, Cuernavaca, Morelos, Mexico, E-mail: javier.idrovo@insp.mx

In a large cohort study published in *Environmental Health Perspectives*, Brenner et al. (2011) confirmed previous results on I-131 exposure and thyroid cancer among a Ukranian population. According to the authors, one motivation to study this association was based on evidence from ecological studies (Jacob et al. 1999) with two methodological limitations: use of grouped doses and poor control of confounding. With these new findings, evidence from ecological, case–control, and cohort studies are consistent; thus, an interesting question is whether there was an ecological fallacy.

**Figure 1:**
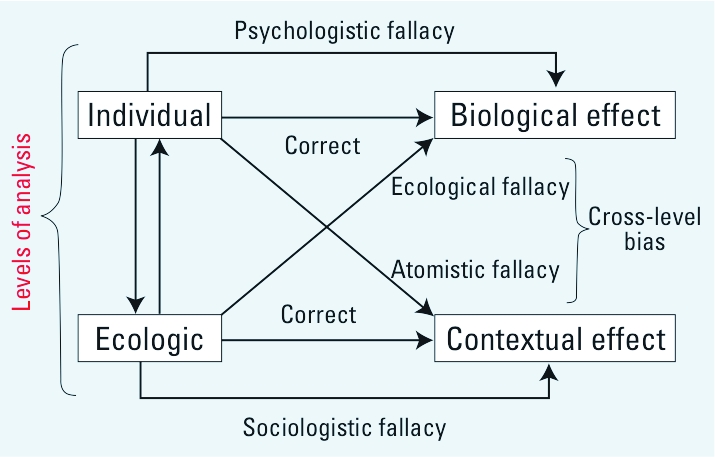
Levels of analysys in epidemiologic studies and potential fallacies during casual inference.

Although ecological studies are important to epidemiology (especially in environmental and social epidemiology), public health practitioners seem afraid of ecological studies. It is a common practice to assume the presence of ecological fallacy (Robinson 1950) and low-level validity when analyzing an ecological study. Most epidemiologists prefer an exclusive individualistic approach, although the importance of a multilevel causal approach is widely recognized (Diez-Roux 2002). In this sense, some authors suggest that it is as important to recognize the presence of ecological fallacy as to recognize psychologistic or individualistic fallacy (Subramanian et al. 2009) (Figure 1).

Thus, it is necessary to have clear guidelines on when there is or not an ecological fallacy. In this sense, I propose three criteria for the identification of ecological fallacy; all three of these should be present to confirm its existence:

- Results must be obtained with ecological (population) data.
- Data must be inferred to individuals. One use of ecological studies is to explore  individual-level association when individual data are not available. When the focus of the study was contextual or based on population effects and there is no inference to individuals, ecological fallacy is not possible. When only the first two criteria are present—which is insufficient to affirm ecological fallacy—it is appropriate to acknowledge that there is a possible relationship and that further study is required.
- Results obtained with individual data are contradictory.

Only when empirical data are available is it possible to confirm that an ecological fallacy is present.
